# Comparative expression analysis in three Brassicaceae species revealed compensatory changes of the underlying gene regulatory network

**DOI:** 10.3389/fpls.2022.1086004

**Published:** 2023-01-04

**Authors:** Jessica Pietsch, Anna Deneer, Christian Fleck, Martin Hülskamp

**Affiliations:** ^1^ Botanical Institute, Biocenter, Cologne University, Cologne, Germany; ^2^ Biometris, Department of Mathematical and Statistical Methods, Wageningen University, Wageningen, Netherlands; ^3^ Spatial Systems Biology Group, Center for Data Analysis and Modeling, University of Freiburg, Freiburg, Germany

**Keywords:** evolution, trichomes, genetic analysis, patterning, brassicaceae, *arabis alpina*, *cardamine hirsuta*

## Abstract

Trichomes are regularly distributed on the leaves of Arabidopsis thaliana. The gene regulatory network underlying trichome patterning involves more than 15 genes. However, it is possible to explain patterning with only five components. This raises the questions about the function of the additional components and the identification of the core network. In this study, we compare the relative expression of all patterning genes in A. thaliana, A. alpina and C. hirsuta by qPCR analysis and use mathematical modelling to determine the relative importance of patterning genes. As the involved proteins exhibit evolutionary conserved differential complex formation, we reasoned that the genes belonging to the core network should exhibit similar expression ratios in different species. However, we find several striking differences of the relative expression levels. Our analysis of how the network can cope with such differences revealed relevant parameters that we use to predict the relevant molecular adaptations in the three species.

## 1 Introduction

Evolutionary differences and adaptive strategies within plants are driven by the structure and function of the underlying gene regulatory networks (GRNs) (Alvarez-Buylla et al. (2007); Mejia-Guerra et al. (2012); Jones and Vandepoele (2020)). Even minute changes in a GRN can result in striking differences between species (Guo and Amir (2021); Schember and Halfon (2022)). In evolutionary developmental approaches, such differences are studied in order to gain insight into the genetic basis of phenotypic diversity (Purugganan (1998); Simpson (2002); Fernandez-Mazuecos and Glover (2017)). A system that is well-suited for such an approach is trichome patterning in *Arabidopsis thaliana* and other Brassicaceae species (Hülskamp (2004); Doroshkov et al. (2019); Chopra et al. (2019)). Genetic analysis of trichome patterning in *A. thaliana* has revealed a complex GRN that controls the regular distribution of trichomes in the leaf epidermis (Marks et al. (1991); Hülskamp et al. (1994); Pattanaik et al. (2014)). Most of the genes found in *A. thaliana* are present in *Arabis alpina* and appear to have the same function in regulating trichome patterning (Chopra et al. (2014)). This suggests that the core of the GRN found in *A. thaliana* might be operating in other Brassicaceae as well. Therefore, the evolutionary analysis of trichome patterning in different Brassicaceae species may enable the identification of subtle changes of the underlying GRN.

In *A. thaliana*, trichomes are initiated in a regular pattern early in leaf development. Genetic analysis identified mutants in which regular pattern formation was disturbed and subsequent molecular analysis revealed the relevant genes (Hülskamp et al. (1994)). One group of genes promotes trichome formation and a second group inhibits trichome formation (Pesch and Hülskamp (2009)). The core of the network is a group of three genes, the R2R3MYB protein encoding gene *GLABRA1 (GL1)* (Oppenheimer et al. (1991); Zhang et al. (2003); Kirik et al. (2005)), the bHLH protein encoding gene *GLABRA3 (GL3)* (Payne et al. (2000); Zhang et al. (2003); Feller et al. (2011))and the WD40 protein encoding gene *TRANSPARANT TESTA GLABRA1 (TTG1)* (van Nocker and Ludwig (2003); Zhao et al. (2008); Zhang and Schrader (2017)). The respective proteins form a complex in which GL1 and TTG1 both bind to GL3 (Payne et al. (2000); Pesch et al. (2015)). This so-called MBW (**M**YB, **b**HLH, **W**D40) complex promotes trichome development (Payne et al. (2000)). In addition, *MYB23* and *EGL3* were found to act redundantly with *GL1* and *GL3*, respectively (Kirik et al., 2001; Kirik et al. (2005)); Zhang et al. (2003)). A second group of genes act as inhibitors of trichome formation. These are all encoded by small R3MYB transcription factors including *TRIPTYCHON (TRY)* (Schellmann et al. (2002); Zimmermann et al. (2004); Wang et al. (2008); Pesch and Hülskamp (2011)), *CAPRICE (CPC)* (Wada et al. (1997); Schellmann et al. (2002)), *ENHANCER OF TRY and CPC1*, *2* and *3 (ETC1, ETC2, ETC3)* (Kirik et al. (2004); Kirik et al., 2005); Tominaga et al. (2008); Wester et al. (2009)) and *TRICHOMELESS1* and *2* (*TCL1* and *TCL2*) (Wang et al. (2007); Gan et al. (2011)). TRY and CPC seem to be the major players as the single mutants exhibit clear phenotypes which is enhanced in combinations with the others suggesting redundant action (Schellmann et al. (2002)). These inhibitors repress the function of the MBW complex by competing with GL1 for binding to GL3/EGL3. The detailed analysis of the function of these genes led to two principles that can explain the generation of trichome spacing patterns without pre-existing information (*de novo* patterning). In short, the first principle is an activator inhibitor model (Meinhardt and Gierer (2000)): the three MBW proteins activate the expression of the inhibitors, that can move within the tissue and repress the MBW function (Digiuni et al. (2008); Pesch et al. (2015)). The second principle is an activator depletion model (Meinhardt and Gierer (2000)). Here, the activator TTG1 is mobile and captured by GL3 in trichome precursors, which in turn leads to a depletion of TTG1 in the neighbouring cells and thereby inhibition of trichome formation (Bouyer et al. (2008); Balkunde et al. (2011)). It is likely that both principles act in parallel (Balkunde et al. (2020)). Mathematical models have been developed to study the behaviour of these principles in more detail (Bouyer et al. (2008); Digiuni et al. (2008); Benitez et al. (2008); Balkunde et al. (2020)). These principles can explain how a trichome cell is selected. In the selected trichome cell, the homeobox transcription factor gene *GLABRA2 (GL2)* is turned on and considered to initiate the differentiation into a trichome cell (Szymanski et al. (1998); Morohashi et al. (2007); Wang and Chen (2008)).

The systematic forward genetic screen for trichome mutants in *A. alpina* has enabled the identification and functional characterization of trichome patterning genes in this species (Stephan et al. (2019); Chopra et al. (2019)). *A. alpina* diverged from *A. thaliana* between 26 and 40 million years ago (Koch et al. (2006); Beilstein et al. (2010); Willing et al. (2015)). At this evolutionary distance it was possible to identify the gene orthologs to those in *A. thaliana* by synteny on the chromosomes (Chopra et al. (2019)). It was therefore possible to unambiguously recognize not only the homologous genes, but also that two of the seven inhibitor genes, *TCL1* and *ETC2*, are missing. The genetic analysis revealed two interesting changes in the GRN. First, the *GL3* gene in *A. alpina* does not appear to act redundantly with *EGL3*. While in *A. alpina* the *gl3* single mutant is completely devoid of trichomes, it requires the *gl3 egl3* double mutant in *A. thaliana* to express the full phenotype (Chopra et al. (2019)). Other than that, the structure of the GRN in *A. alpina* seems to the same as in *A. thaliana*. It is, however, noteworthy, that the response of the network to overexpression of *GL3* is very different such that this causes the production of more trichomes in *A. thaliana* and less in *A. alpina*. This difference in the behaviour can be explained by different relative expression levels of two key genes, *GL1* and *TRY*, in the two species (Chopra et al. (2019)). Modelling revealed that this change in the parameters is sufficient to explain the different responses to *GL3* overexpression.

In this work, we compared the relative expression levels of trichome patterning genes in *A. thaliana*, *A. alpina* and *Cardamine hirsuta*. The additional species *C. hirsuta* is estimated to have diverged between 13 and 43 million years ago from *A. thaliana* (Koch et al. (2001); Beilstein et al. (2008)). For comparison between the species, the expression levels of the trichome patterning genes were considered relative to *GL3/EGL3* as all patterning proteins bind to GL3 thereby regulating its activity. We observed striking differences raising the question whether and how the GRN established in *A. thaliana* has adapted to this. We analysed the differences by mathematical modelling and determined which parameters (i.e. interactions and regulations in the GRN) could explain the observed differences in the relative expression levels.

## 2 Methods

### 2.1 Primer establishment and validation

qPCR primers must meet particular requirements. Preferably intron-spanning primers were designed using GenScript Real-time PCR Primer Design (www.genscript.com) with an optimal melting temperature of 60±2°C and sequence specific amplicons of ideally 150-200 bp. They exhibit one single band of the expected size in agarose gel electrophoresis and a single peak in the melting curve. Amplification efficiency and correlation were determined based on serial cDNA dilution steps (1:10, 1:20, 1:40,1:80, 1:160, 1:320). Cq and log10 values of the dilution series were used to calculate the slope Δ by


(1) $$\text{Δ}=\frac{\sum_{i=1}^{N}\left(x_{i}-\bar{x}\right)(y_{i}-\bar{y})}{\sum_{i=1}^{N}{\left(x_{i}-\bar{x}\right)}^{2}}$$

where *N* is the number of dilution steps. The slope served to calculate the primer efficiency *E* by


(2) $$E=100\cdot \left(10^{\frac{-1}{\Delta }}-1\right)$$

The *R* ^2^ correlation of the Cq and the log10 values was calculated using


(3) $$\rho_{x,y}=\frac{Cov\left(X,Y\right)}{\sigma_{x}\sigma_{y}}$$

Amplification efficiencies of 100% ± 20 for genes of interest and 100% ±10 for reference genes as well as a linear standard curve with a correlation of ≥0.99 were accepted. Sequences were taken from TAIR (www.arabidopsis.org), from Genomic resources for *Arabis alpina* (www.arabis-alpina.org) and from *Cardamine hirsuta* genetic and genomic resource (http://chi.mpipz.mpg.de).

### 2.2 Plant material and sample preparation

For this analysis we used *Arabidopsis thaliana* Col-0, *Arabis alpina* Pajares and *Cardamine hirsuta* Ox. Cotyledons as well as juvenile leaves (leaf one and two for *Arabidopsis thaliana* and *A. alpina*, additionally leaf three for *C. hirsuta*) of plant seedlings were removed to gather 200-400 *μm* sized leaves with on-going trichome patterning machinery. *A. thaliana* plants were 10 days old, *A. alpina* 14 days and *C. hirsuta* 7 days. Material of up to 45 plants was collected per biological replicate, frozen in liquid nitrogen and stored at −80 C until further processing. RNA extraction was performed using the Tri-Reagent method including DNaseI treatment and quality control was ensured *via* bleach gel and photometry. cDNA synthesis was carried out according to the manufacturer’s protocol (RevertAid First Strand cDNA Synthesis Kit; Thermo Fisher Scientific) using 1.5 *μg* RNA per sample because pre-tests had revealed that 1 *μL* undiluted cDNA based on 1 *μg* RNA were required to obtain Cq values<30. qPCR protocols were standardized using three biological as well as three technical replicates, master mixes and always both reference genes on each plate.

### 2.3 Analysis of qPCR data

A two-sided Grubbs test (α=0.05) was performed to identify outliers. Normalization of the data was conducted according to the geNorm manual (Vandesompele et al. (2002)), describing gene expressions relatively to each other. Special considerations are given to normalization factors and the individual primer efficiencies *∈*. Thereby not a generalized gene duplication per cycle (1 + 1) is assumed, but the individual amplification rate (1+*∈*) is used for further calculations. The expression data of each species was normalized by two different reference genes. Using the variability of the reference genes and not the Cq values, allows interspecies comparison even with different reference genes for each organism.

### 2.4 Compiling GL1 synteny

Arabidopsis thaliana was used as reference to elaborate the synteny of *GL1* comparing it with *A. alpina* and *C. hirsuta*. The *AtGL1* sequence was used to perform a BLAST search against the *C. hirsuta* CDS database (http://bioinfo.mpipz.mpg.de/blast/cgi-bin/public_blast_cs.cgi). More than a dozen of loci spanning the first three highest ranked genes were blasted against *Arabidopsis thaliana*. The *AaGL1* ortholog as well as its adjoining genes were identified using the 1x1 orthologs table from the Arabis alpina website (http://www.arabis-alpina.org/data/ArabisAlpina/data/Aa_At_ortho_1x1.txt)

### 2.5 Mathematical modelling

The model consists of 8 components, which are modelled in the form of a system of coupled ordinary differential equations. These components include the proteins TTG1, GL1, GL3, TRY, CPC and ETC. Note that the species designated by GL1 and ETC are assumed to be the combined behavior of GL1, MYB23 and ETC1, ETC2 and ETC3, respectively. Additionally, the complex formation between GL3 and TTG1 and GL3 and GL1 is explicitly modelled, whereas the binding between GL3 and the inhibitors TRY, CPC and ETC is implicitly modelled since these do not feed back into the system. This model consists of 31 parameters that describe processes such as degradation, binding, activation and transport. This model is based on previously published versions and is extended by the inclusion of ETC (Digiuni et al. (2008); Bouyer et al. (2008); Chopra et al. (2019); Balkunde et al. (2020)). The system of equations is


(4) $$\partial_{t}[TTG1{]}_{j}=\;\theta_{1}-[TTG1{]}_{j}(\theta_{2}+\theta_{3}\left[GL3{]}_{j}\right)+\theta_{2}\theta_{4}\hat{L}{\left[TTG1\right]}_{j}$$

(5) $$\partial_{t}[GL1{]}_{j}=\;\theta_{5}+\theta_{6}[AC2{]}_{j}-{\left[GL1\right]}_{j}(\theta_{7}+\theta_{8}\left[GL3{]}_{j}\right)+\theta_{7}\theta_{30}\hat{L}{\left[GL1\right]}_{j}$$

(6) $$\begin{array}{l} \partial_{t}[GL3{]}_{j}=\;\theta_{9}+\frac{\theta_{10}\theta_{11}{\left[AC1\right]}_{j}^{2}}{\theta_{11}+{\left[AC1\right]}_{j}^{2}}+\frac{\theta_{12}\theta_{13}{\left[AC2\right]}_{j}^{2}}{\theta_{13}+{\left[AC2\right]}_{j}^{2}}-\\ {}[GL3{]}_{j}(\theta_{14}+\theta_{3}[TTG1{]}_{j}+\theta_{8}[GL1{]}_{j}+\theta_{15}[TRY{]}_{j}+ \end{array}$$

(7) $$\theta_{16}[CPC{]}_{j}+\theta_{17}[ETC{]}_{j})+\theta_{14}\theta_{31}\hat{L}{\left[GL3\right]}_{j}$$

(8) $$\begin{array}{l} \partial_{t}[TRY{]}_{j}=\;\theta_{18}[AC1{]}_{j}^{2}-[TRY{]}_{j}(\theta_{19}+\theta_{15}\left[GL3{]}_{j}\right)+\\ \theta_{19}\theta_{20}\hat{L}[TRY{]}_{j} \end{array}$$

(9) $$\begin{array}{l} \partial_{t}[CPC{]}_{j}=\;\theta_{21}[AC2{]}_{j}^{2}-[CPC{]}_{j}(\theta_{22}+\theta_{16}\left[GL3{]}_{j}\right)+\\ \theta_{22}\theta_{23}\hat{L}[CPC{]}_{j} \end{array}$$

(10) $$\begin{array}{l} \partial_{t}[ETC{]}_{j}=\;\theta_{24}[AC1{]}_{j}^{2}+\theta_{25}[AC2{]}_{j}^{2}-[ETC{]}_{j}(\theta_{26}-\theta_{17}\left[GL3{]}_{j}\right)+\\ \theta_{26}\theta_{27}\hat{L}[ETC{]}_{j} \end{array}$$

(11) $$\partial_{t}[AC1{]}_{j}=\;\theta_{3}[GL3{]}_{j}[TTG1{]}_{j}-\theta_{28}[AC1{]}_{j}$$

(12) $$\partial_{t}[AC2{]}_{j}=\;\theta_{8}[GL3{]}_{j}[GL1{]}_{j}-\theta_{29}[AC2{]}_{j}$$

where $\hat{L}$ indicates the coupling equation between cells, given by


(13) $$\begin{array}{l} \hat{L}[\chi {]}_{x,y}=[\chi {]}_{y-1,x}+[\chi {]}_{y+1,x}+[\chi {]}_{y,x-1}+[\chi {]}_{y,x+1}\\ +[\chi {]}_{y+1,x-1}+[\chi {]}_{y-1,x+1}-6[\chi {]}_{y,x}. \end{array}$$

for any species *χ* and cell at coordinates (*x, y*). Patterns were simulated on a grid of 20-by-20 cells with hexagonal connectivity on a domain with zero-flux boundary conditions. The initial conditions are given by the steady state of a single-cell model (i.e. $\hat{L}=0$ ) plus small inhomogeneous perturbations, sampled from the standard uniform distribution. The trichomes on the grid are identified by cells that have relatively high amounts of active complex (AC1 + AC2), specifically, cells that have more than the half-maximum of total AC are designated as trichomes. The cluster density of trichomes was averaged over 10 simulations, each with randomized initial conditions. Parameter sets that produced less than 10% clusters are used for further analysis.

Given that we are only interested in parameter sets that form patterns, we apply linear stability anaylsis to identify these sets (Murray (2001)). In the domain of interest, a diffusion-driven instability (Turing instability) occurs (Turing (1952)), resulting in an inhomogeneous patterning state. In linear stability analysis, the stability of a uniform steady state is verified by determining whether effects of small perturbations to the ODE system decay over time. Turing instability was tested by the following criteria: starting from a uniform steady state (i) the steady state in the absence of diffusion is stable and (ii) the steady state in the presence of diffusion is unstable (Murray (2001)). For criterion i this means this means that all eigenvalues of the Jacobian of the system in (4) - (12) evaluated at steady state must be negative. To perform the same test for criterion ii we decoupled the system by Fourier transformation and analysed the eigenvalues (Bouyer et al. (2008); Digiuni et al. (2008); Balkunde et al. (2020)), where the real part of at least one of the eigenvalues must be positive.

### 2.6 Parameter estimation

The previous section describes the rationale and rules for the parameter sets used for the model. To use the model for our qPCR data set we estimated the parameter sets through an optimization routine where the qPCR data are used in a cost function. The goal is to arrive at a distribution of values for these parameters for each of the species. Note that this problem suffers from non-identifiability (Kreutz et al. (2013)), i.e., no unique value or bounded confidence interval can be determined for the parameters; for this, additional data would be required that is simply not available. Nonetheless, through a multi-start optimization routine that ensures multiple optimal solutions, it is possible to deal with the uncertainty in the system and arrive at predictions about possible genetic adaptations on a regulatory level that differentiates the three species from each other.

The analysis used here requires solving a constrained multivariable minimization problem (Boese et al., 1994). Specifically, we aim to find the minimum of the problem specified by


(14) $$\underset{\theta }{\min}f\left(\theta \right)\;\text{such that}\;\{\begin{array}{l} c\left(\theta \right)\geq 0\\ \text{lb}\leq \theta \leq \text{ub} \end{array}$$

where *c*(***θ***) is a non-linear constraint function, *f*(***θ***) is a scalar cost-function and lb and ub are the lower- and upper-bounds of the parameter vector ***θ***. The cost function *f* is a normalized sum-of-squares given by


(15) $$f\left(\theta \right)=\sum_{i=1}^{N}\frac{{\left({\bar{y}}_{i}\left(\theta \right)-y_{i}\right)}^{2}}{y_{i}^{2}}$$

where ${\bar{y}}_{i}\left(\theta \right)$ is the expression level of the i-th gene out of *N* total genes predicted by the model and *y_i_* is the corresponding datapoint. Given that the model simulates the concentration in a tissue, ${\bar{y}}_{i}$ (***θ***) is the average of gene *i* across the tissue, relative to the sum of GL3 and EGL3, similar as the data.

The constraint function *c*(***θ***) is chosen such that the parameters ***θ*** fall in the Turing Space, i.e. are capable of patterning. To achieve this, we make use of linear stability analysis as described above and determine the eigenvalues of the Jacobian of the system of equations. *c*(***θ***) is given by


(16) $$c\left(\theta \right)=\text{Re}\left(\lambda_{\text{max}}\right)$$

where *λ*_max_ is the largest eigenvalue of the Jacobian. By determining whether the real part of the largest eigenvalue is positive (i.e. *c*(***θ***) ≥ 0), we learn that the parameter set ***θ*** can form a pattern, which constrains the allowable range of parameters. Note that this range is also constrained by the choice of bounds (lb and ub) of the optimization problem. In this case, we set the interval for the each of the parameters in ***θ*** to [0.01,100], to allow a range of multiple orders of magnitude. One further constraint, which is applied in post-processing, is that the pattern produced by ***θ*** must not show any clusters of trichomes, as is the case in all the patterns formed by the three species. As such, we limit the range of parameter values to those that simulate a realistic pattern and produce the best possible fit to the data according to *f*(***θ***). Finally, the optimization problem is started from multiple, randomly generated initial points. This set of initial points is generated by a Sobol sequence to ensure a good coverage of the parameter space (Sobol (2001)). Starting from these randomly generated initial points, the optimization converges to a local minimum that satisfies the constraints, leading to a distribution of optimal parameter sets $\hat{\theta }$ that correspond to the local minima. This procedure is followed until 100 optimized parameter sets are obtained for each of the species.

These distributions are then used in a statistical analysis to determine which of the parameter distributions are statistically different between the species. Towards this end, we use the two-sample Kolmogorov-Smirnov (KS) goodness-of-fit hypothesis test to determine if two empirical distributions are drawn from the same (unknown) underlying population cumulative distribution functions (Stephens (1974)).

### 2.7 Sensitivity analysis

The sensitivity of the trichome density to the each of the individual parameters is determined, using a variation on the elementary effects (EE) test (Campolongo et al., 2007; Saltelli, 2008). The EE is a one-at-a-time screening method, i.e.,only one parameter is varied at a time and the variation in the output is measured (Campolongo et al. (2007); Saltelli (2008)). For a model with *N* parameters, each parameter *θ_i_*,*i* = 1,…*N*, is assumed to vary across *p* selected levels in the parameter space. The region of experimentation Ω is an *N*-dimensional *p*-level grid. In standard sensitivity practices, parameters are assumed to be uniformly distributed in [0,1] and then transformed from the unit hypercubeto their known distributions (Saltelli (2008)). In this case, we adapt this region to ensure that the parameters fall within the Turing Space. The lower limit of Ω is by default chosen to be 10^-1^ and the upper limit 10,where every point Δ in the grid is the perturbation applied to *θ_i_* for which the *EE_i_* is determined. In the case that either limit would shift ***θ*** outside of the Turing space, then the lower limit is adjusted to the smallest value between [10^-1^,1] that according to linear stability analysis falls within the Turing space, and the upper limit is the largest value between [1,10] that falls within the Turing space. This means that the *p*-level grid in Ω can have different upper and lower limits, depending on the allowable range according to linear stability analysis, but always consists of the same number of grid-points. Furthermore, these grid-points are chosen such that they are logarithmically spaced.

For the trichome patterning model, we have *N* = 31 and choose *p* = 10. We perform the EE sensitivity analysis for the top 10 best-fitting parameter sets $\hat{\theta }$ resulting from the optimization routine. For a given set ${\hat{\theta }}^{k},\;k=1,\ldots ,10$ the EE of the i-th parameter is defined as:


(17) $${\text{EE}}_{i}\left({\hat{\theta }}^{k}\right)=\frac{Y\left(\theta_{1},\ldots ,\theta_{i-1},\theta_{i},\theta_{i+1},\ldots ,\theta_{N}\right)-Y\left({\hat{\theta }}^{k}\right)}{\theta_{i}\cdot \Delta -\theta_{i}}$$

where Δ is a value in the *p*-level grid with the limits chosen as described above. Then, the absolute values of the EE*_i_*, computed at p different grid points, are averaged to get


(18) $${\text{E}\bar{\text{E}}}_{i}=\frac{1}{p}\sum_{j=1}^{p}|{\text{EE}}_{i}^{j}|.$$

Finally, we average over all ${\text{E}\bar{\text{E}}}_{i}$ for every ${\hat{\theta }}^{k}$ .

## 3 Results

### 3.1 Comparison of trichome gene expression in different species

All three species considered here, *A. thaliana*, *A. alpina* and *C. hirsuta* produce regularly spaced trichomes on leaves (Greese et al. (2012); Chopra et al. (2019)). The trichome density differs such that *C. hirsuta* has a lower density, and *A. alpina* a higher density as compared to *A. thaliana*. A meaningful quantitative comparison of trichome density appears not to be possible as leaf sizes, growth dynamics and the juvenile-to-adult transition differ making it arbitrary to choose the proper mature leaves for comparison. We therefore focused on qualitative and ratiometric comparisons in this study.

A direct comparison of the expression levels of trichome genes between species by qPCR is not possible for various reasons. In particular, the primers are different for the same genes and normalization was done with difference reference genes. We therefore compared the expression levels between species by normalizing the expression to the bHLH genes. The bHLH protein is the central component of the MBW complex to which the activators *TTG1* and R2R3 MYB proteins bind (Payne et al. (2000)) and on which the R3 MYB negative regulators exert their repressive effect by competitive binding with the R2R3 MYBs (Digiuni et al. (2008)). It is conceivable that the outcome of this GRN depends on the concentrations of the other patterning proteins relative to the bHLH. We therefore considered the bHLH expression levels a good reference to judge and compare to the relative changes of all other patterning genes. We combined *GL3* and *EGL3* for the comparison between the species because the two genes act redundantly in *A. thaliana* and have similar molecular roles in trichome patterning (Zhang et al. (2003); Bernhardt et al. (2003); Morohashi et al. (2007)).

Another aspect to enable a comparison between the species is the choice of plant material. For our qPCR experiments we used young leaves at developmental stages in which trichome patterning was still ongoing as recognized by the presence of incipient and young stages of trichome development at the base of leaves. These stages could be unambiguously identified in all three species.

### 3.2 Relative trichome gene expression differs in *A. thaliana, A. alpina* and *C. hirsuta*

In a first step, we identified the bonafide orthologs of the *Arabidopsis* trichome patterning genes in *A. alpina* and *C. hirsuta* by sequence similarity and synteny (**Supplementary Figure S1**). Primers were designed to meet the Minimum Information for Publication of Quantitative Real-Time PCR Experiments (MIQE) guidelines (Bustin et al. (2005)).

**Figure 1:**
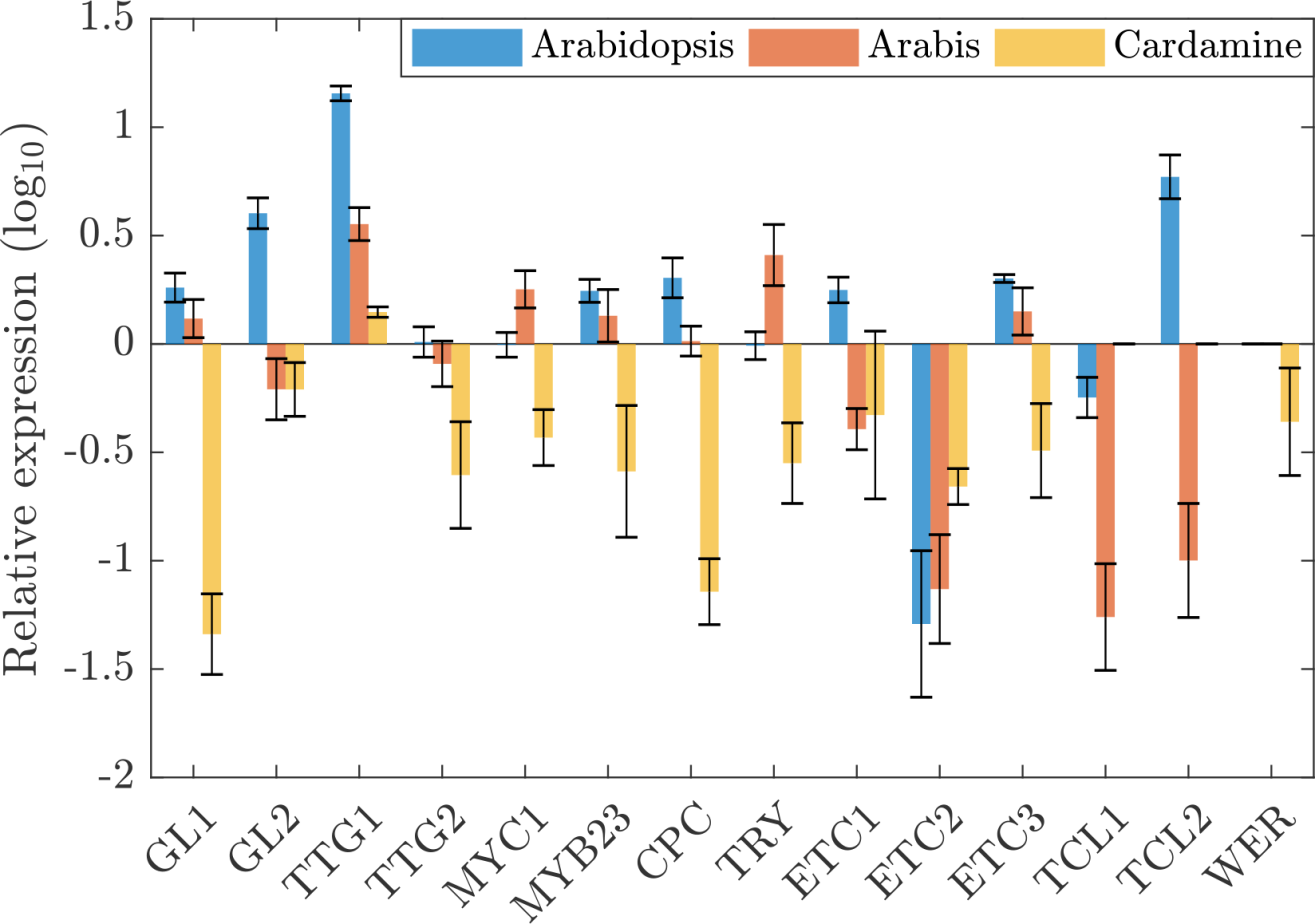
Comparative patterning gene expression. Depicted are the expressions and fold changes of 15 patterning genes in *A. thaliana* (blue), *A. alpina* (red), and *C. hirsuta* (yellow) relative to the sum of GL3 and EGL3 in the respective species.

Plants were grown on soil and young leaves were harvested at stages at which incipient developing trichomes were seen. The first two leaves and the cotyledons were removed. Quantitative Real-Time PCR experiments were done with three biological replicas and normalized to a set of species-specific reference genes. To enable a comparison between species we normalized all expressions with *GL3/EGL3*. **Figure 1** shows the relative expression levels of patterning genes normalized to the combined transcript levels of *GL3* and *EGL3* which was set to one (see also **Table S1**). In *A. thaliana*, *GL3/EGL3* appear to be the limiting factors among the other trichome activating genes (**Figure 1**). *AtTTG1* expression is about 14-fold higher and the expression of *AtGL1* and *AtMYB23* both show an about 2-fold higher expression. Consistent with the previous finding that *AtGL1* and *AtMYB23* act redundantly in *Arabidopsis* (Kirik et al., 2001), they show a similar expression level and we combined their expression for the modelling approach to reduce the complexity (see below). The relative expression levels of the inhibitors were different with *AtCPC, AtETC1, AtETC3* and *AtTCL2* being higher and *AtETC2* and *AtTCL1* lower than *AtGL3/AtEGL3*.

The expression profile in *A. alpina* is fairly similar to that in *A. thaliana*. In *C. hirsuta* we found a strikingly different pattern of the relative expression of trichome patterning genes. Here, most of the patterning genes exhibit lower expressions as compared to *ChGL3/ChEGL3*. In particular, the expression of *ChGL1* and *ChCPC* were drastically lower as compared to the other two species.

### 3.3 GL1, MYB23 and WER expression differs in *Arabidopsis thaliana*, and *C. hirsuta*

**Figure 2:**
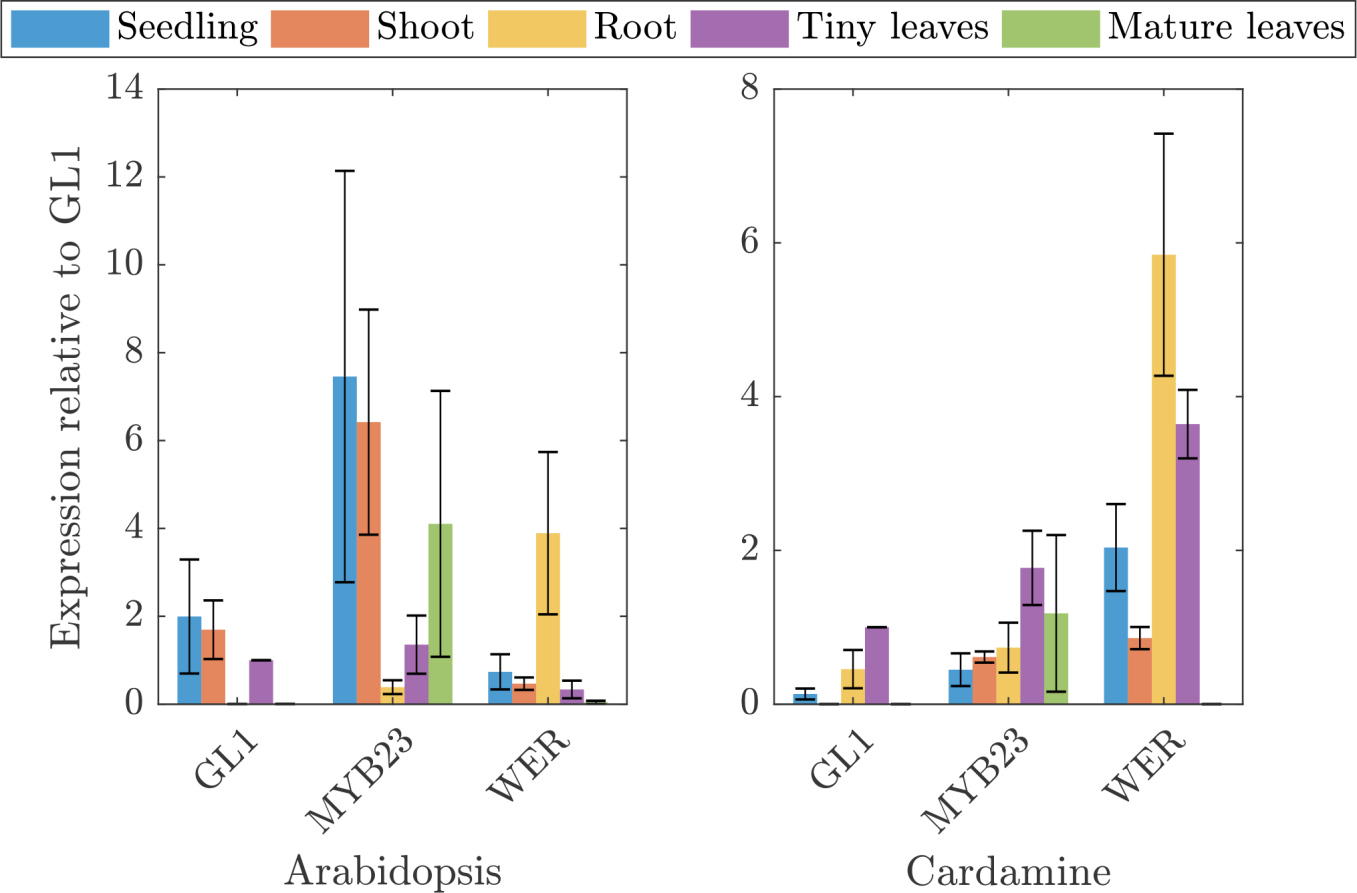
Quantitative expression analysis of three MYB homologs in *A. thaliana* and *C. hirsuta* in different tissues. Depicted is the expression of *GL1, MYB23*, and *WER* in *Arabidopsis thaliana* and *Cardamine hirsuta* in seedlings (blue), shoots (red), roots (yellow), tiny leaves (purple), and mature leaves (green), relative to GL1 expression in tiny leaves of the respective species.

The very low relative and also absolute expression levels of *ChGL1* in *C. hirsuta* raised the question whether the function of *ChGL1* is redundantly provided by *ChMYB23* (Kirik et al. (2001)) or even *ChWER* (Lee and Schiefelbein (1999)). To study this in more detail, we compared the expression of the three genes in five different tissues between *A. thaliana* and *C. hirusta* (**Figure 2**). To facilitate a comparison in the context of trichome patterning, we normalized the expression levels with respect to the *GL1* expression in young leaves. As expected, *AtGL1* and *AtMYB23* are expressed in most aerial tissues in *Arabidopsis thaliana* but not in the root, whereas *AtWER* expression was detected strongly in the root. In *C. hirsuta*, *ChGL1* and *ChMYB23* expression was absent or low in all tissues. Surprisingly, *ChWER* expression was not only high in roots, but also in young leaves. Here, *ChWER* expression was 2.6 fold higher than that of *ChGL1*. These findings suggest that the tissue specific functions of *GL1, MYB23* and *WER* might be different in the species. Given that *AtGL1* and *AtWER* proteins have equivalent function during trichome initiation in *A. thaliana* (Lee and Schiefelbein (2001); Kirik et al. (2005)), it is conceivable that the higher expression of *ChWER* can substitute the low levels of *ChGL1*.

### 3.4 Modelling to predict the molecular adaptations to relative expression differences between the three species

**Figure 3:**
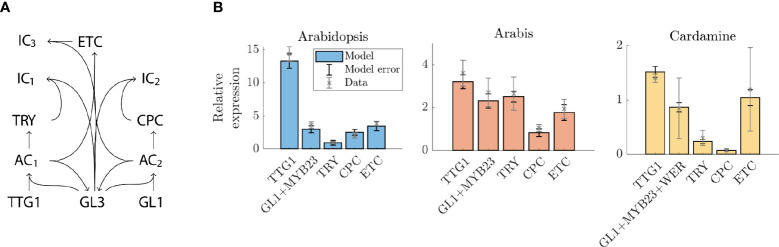
Expression levels in the model compared to qPCR data. **(A)** Schematic representation of model network. AC1 and AC2 are the active complexes TTG1 GL3 and GL1 GL3; IC1 and IC2 are the inactive complexes TRY GL3 and CPC GL3. **(B)** Expression levels of genes in *A*. *thaliana, A. alpina*, and *C*. *hirsuta* relative to GL3+EGL3. The qPCR data is indicated by grey crosses with error bars (representing biological replicates) and the model expression levels are indicated by the bars (blue for *A*. *thaliana*, red for *A*. *alpina*, yellow for *C*. *hirsuta*), the model error (black error bars) is the error over the 10 best fitting parameter sets.

The functional comparison of the regulation between *A. thaliana* and *A. alpina* suggests that the core of the underlying regulatory network of trichome patterning is conserved (Chopra et al. (2019)). Consistent with this, all relevant orthologs of the relevant *A. thaliana* genes are also found in *C. hirsuta*. The qPCR data show striking differences in the relative expression levels. Given that trichome initiation is driven by the activity of the MBW complex, in which the components undergo competitive binding, it is surprising that the GRN can tolerate such differences (Digiuni et al. (2008); Pesch et al. (2015)). We used mathematical modelling to explore whether the *Arabidopsis*-based GRN is capable of coping with such differing relative expression patterns. And if so, which parameters can compensate for changes in the relative expression levels and to which of these is the pattern most sensitive? Towards this end, we developed a model based on previous versions (Bouyer et al. (2008); Digiuni et al. (2008); Balkunde et al. (2020)) and consisting of *TTG1, TRY, CPC, ETC1/ETC2/ETC3, GL3/EGL3* and *GL1/MYB23/WER* (**Figure 3A**). Note that the GRN that is modelled is the same for each species (**Figure 3A**) and that the difference between the species comes from differences in the underlying parameters.

We define two criteria for the model to fulfil. First, the parameter set has to reproduce the same relative differences as found for the patterning genes. Second, it has to simulate realistic trichome patterns, i.e., patterns must not show any clustering of trichomes (Greese et al., 2012). We varied all 31 parameters using a non-linear optimization routine such that the model most accurately reproduces the expression data (**Figure 3**). This is surprisingly well possible for the expression data sets of all three species with many different parameter sets.

**Figure 4:**
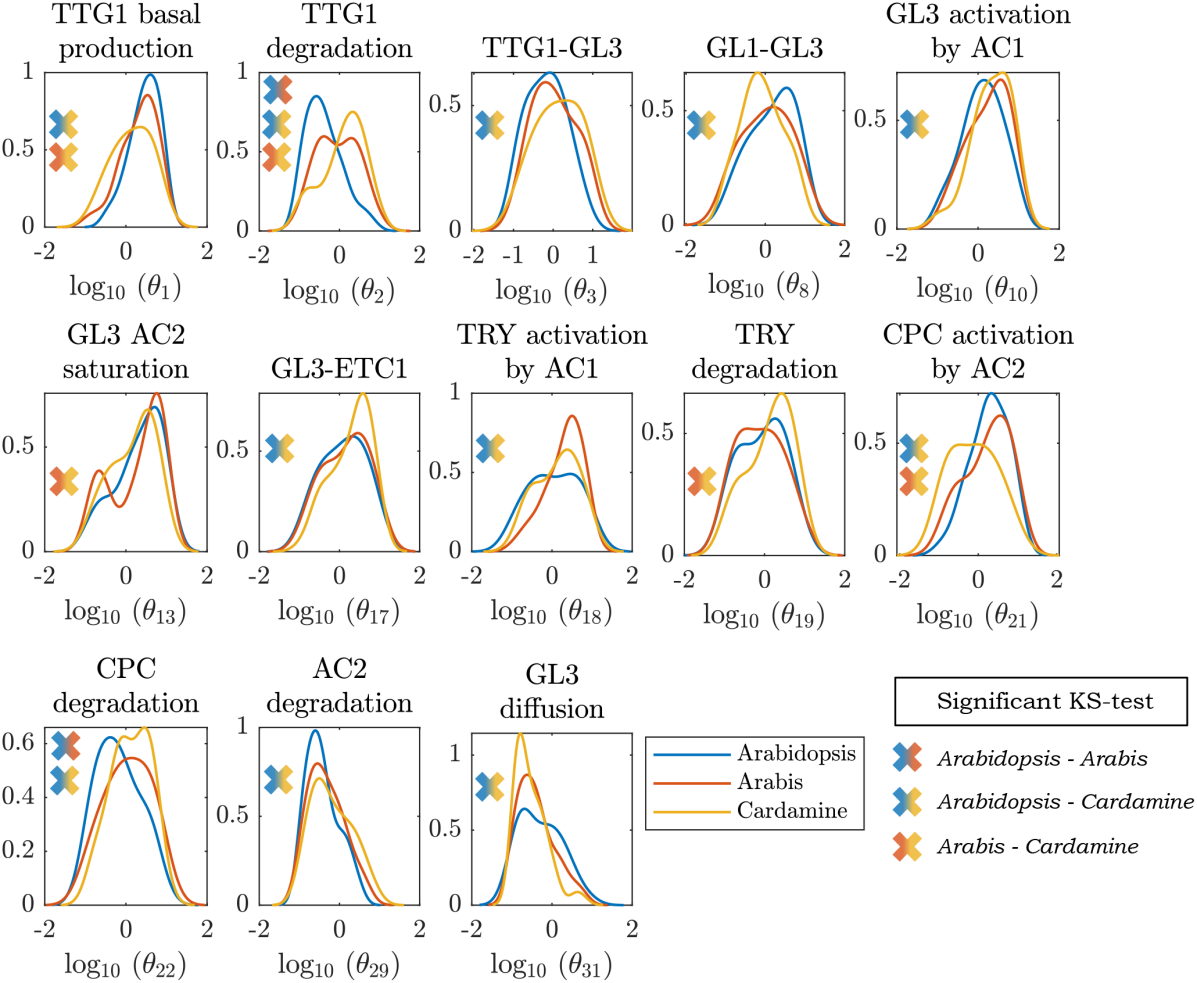
Parameter profile densities. Parameter distributions that differed between the species according to a Kolmogorov-Smirnov test, obtained from fitting the model output to the qPCR data. The crosses indicate between which pair of species the distributions were found to statistically differ. The titles indicate the biological interpretation behind the parameter *θ_i_* on the x-axis.

The identification of a large number of parameter combinations for each species enabled us to compare the distributions of the parameters between the three species in search for striking differences. Towards this end, we used a Kolmogorov-Smirnov test (Stephens (1974)) to identify parameter distributions that are significantly different between the species. Out of the 31 parameters only 13 fulfilled this criterion and were considered parameters that are relevant for compensating different expression ratios in all three species. The distributions are shown in **Figure 4**. The 13 parameters have significantly different distributions for at least two of the species, indicating that the *Arabidopsis*-based model can cope with different relative expression levels by compensatory changes of different parameters. For the other 18 parameters we did not find a significant difference (**Supplementary Figure S2**).

This analysis revealed three interesting differences. First, all parameters regulating the activity of TTG1 in *A. thaliana* differ from the distributions of *A. alpina* and *C. hirsuta* (except for the diffusion rate of TTG1). It is conceivable that this is due to the relatively high expression of *AtTTG1* in *A. thaliana* that could be compensated by parameters changes reducing its activity such as the basal production rate and degradation rate. Second, the parameters regulating *TRY* activity differ between *A. alpina* and the other two species. The model predicts a higher activation rate of *AaTRY* by the complex between GL3 and TTG1 in *A. alpina* that would explain the relatively high levels of *AaTRY* in this species. Third, the regulation of *ChCPC* in *C. hirsuta* differs from the other two species. We found significant differences for the activation of *ChCPC* by the complex between GL3 and GL1 and its degradation rate. Both would compensate for the relative low amount of *ChCPC* expression in *C. hirsuta*. These three cases exemplify the versatility of the trichome patterning network and show how, despite the varying underlying differences between regulatory mechanisms, the same core network is capable of robustly producing a realistic trichome pattern in all three species.

**Figure 5:**
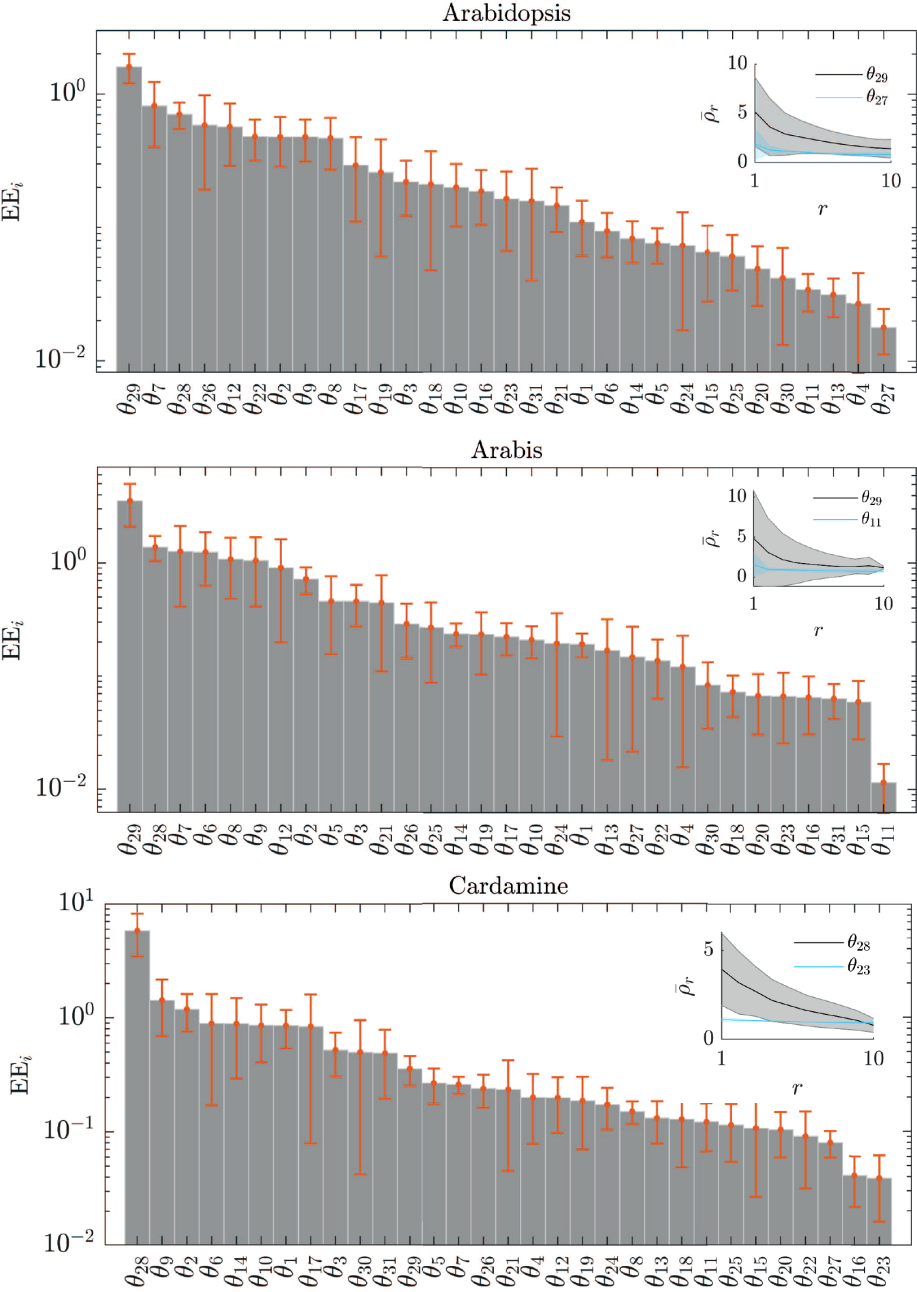
Sensitivity of parameters to trichome density. The elementary effects (EEs) of each of the parameters *θ_i_* in the model indicates the sensitivity of the trichome density to changes in *θ_i_*, sorted by the respective EE value. The error bars indicate the standard deviation in the EE for the ten best-fitting parameter sets. The inset shows the average and the spread (shaded region) of the trichome density ( ${\bar{\rho }}_{r}$ ) for the ten best-fitting parameter sets at each of the ten grid points *r* of the EE test, for the most sensitive parameter (grey) and the least sensitive parameter (blue).

The comparison of parameter distributions provides insight into the adaptability of the network to the different expression levels but does not immediately provide information on the effects on trichome patterning. This is possible by determining the sensitivity of trichome density to changes in each of the parameters in all three species (**Figure 5**). This allows predicitions on which parameter is most influential in patterning and whether this varies between species.

Our sensitivity analysis predicts that for all three species the stability of the TTG1-GL3 complexes (*θ*_28_) is one of the most sensitive parameters. In *A. thaliana* and *A. alpina* the stability of the GL1-GL3 complex (*θ*_29_) and the degradation rate of GL1 (*θ*_7_) are among the most sensitive parameters. In *C. hirsuta*, the basal production of GL3/EGL3 is relevant (*θ*_9_) and the degradation of TTG1 (*θ*_2_). Taken together, the sensitivity analysis predicts that the amount of the active complexes most strongly influences the trichome density and that this is a common feature in all three species. *C. hirsuta* differs in that the role of GL3-TTG1 is more relevant than that of GL3-GL1. For an overview of the biological interpretation of all other parameters in **Figure 5** see **Supplementary Table 2**.

## 4 Discussion

In this study, we have compared the expression levels of trichome patterning genes in the three closely related Brassicaceae species *A. thaliana*, *A. alpina* and *C. hirsuta*. Given that we selected one specific ecotype of each species, our analysis is only a snapshot of possible variation in each of the three species. However, within this limitation, the analysis and comparison of the GRN properties is possible. We aimed to use the variation of the relative expression levels to understand the potential of the GRN underlying trichome patterning. For our mathematical modelling approach, we used a complex model that considers the genetic interactions and simulates concentrations on the protein level to consider differential complex formations (Pesch et al., 2015). The details of transcription and translation are not explicitly modelled as this would add another layer of complexity and thereby more parameters without gaining an extra value in the absence of additional data. This approach enabled us to evaluate the parameter changes with respect to many different aspects of the patterning process including transcriptional regulation, differential complex formation and stability of the transcript/protein. The possible downside is that we have to consider a 31-dimensional parameter space making it necessary to use statistical approaches to monitor the effect of different parameters and their combinations.

What did we learn? First of all, the structure of the GRN network established in *A. thaliana* is sufficient to generate a trichome pattern even if the relative expression levels show an order of magnitude difference. Second, the GRN compensates for differences in the relative expression patterns by changing other parameters. Not all, but only a subset of the parameters is important for this. Third, one type of parameter – the stability of the MBW complexes – is among the most important in all three species. These predictions might be instrumental for future experiments as they help to focus on aspects of the GRN network that have not been studied so far.

A second unexpected result followed from our analysis of the relative transcript levels of the three R2R3MYBs: *GL1, MYB23* and *WER*. The three *Arabidopsis thaliana* genes cluster together in a subgroup of R2R3-MYB and are characterized by a unique amino acid motif near their C-termini, which is not within the MYB domain (Stracke et al. (2001)) and are likely to be the result of gene duplications. *GL1* and *MYB23* act redundantly in the regulation of trichome patterning but have distinct functions in the regulation of trichome branching (Kirik et al. (2005)). Both are only important for trichome formation but not involved in root hair patterning, which is specifically regulated by *WER*. This trait specificity is due to differences in the transcriptional regulation as WER and GL1 are equivalent proteins (Lee and Schiefelbein (2001)). Also, overexpression of *MYB23* can rescue the *wer* mutant phenotype indicating that the protein can substitute WER in this context (Tominaga-Wada et al. (2012)). In *Arabidopsis* we found *AtWER* expression in leaves was detectable, but very low compared to *AtMYB23* and *AtGL1*. When considering that the expression of the three genes is important for their function and that the proteins are functionally similar, it is conceivable that *WER* might have a function in trichome development in *Cardamine*. In fact, by this argument, *WER* would be most important in root and leaf epidermal patterning as it is also most prominently expressed in roots. We therefore hypothesize that our findings reflect the evolutionary sub-functionalization of the three homologous *MYB* genes in trichome and root hair regulation. Functional assays, ideally including mutant analysis in *Cardamine* will be required to test this hypothesis.

## Data availability statement

The original contributions presented in the study are included in the article/**Supplementary Material**. Further inquiries can be directed to the corresponding authors.

## Author contributions

JP planned and did the experimental work and analysis. AD developed the mathematical models and performed the analysis. CF and MH contributed to the design and conception of the study. JP, AD, CF and MH wrote the manuscript. All authors contributed to the article and approved the submitted version.
