# Supplementary material for: Comparative expression analysis in three Brassicaceae species revealed compensatory changes of the underlying gene regulatory network
Source: Front Plant Sci. 2023 Jan 4;13:1086004. doi: 10.3389/fpls.2022.1086004 (PMC9845631; doi:10.3389/fpls.2022.1086004)
Supplement: Supplementary file 1 [file Presentation_1.pdf]

Supplementary Material

1 SUPPLEMENTARY FIGURES

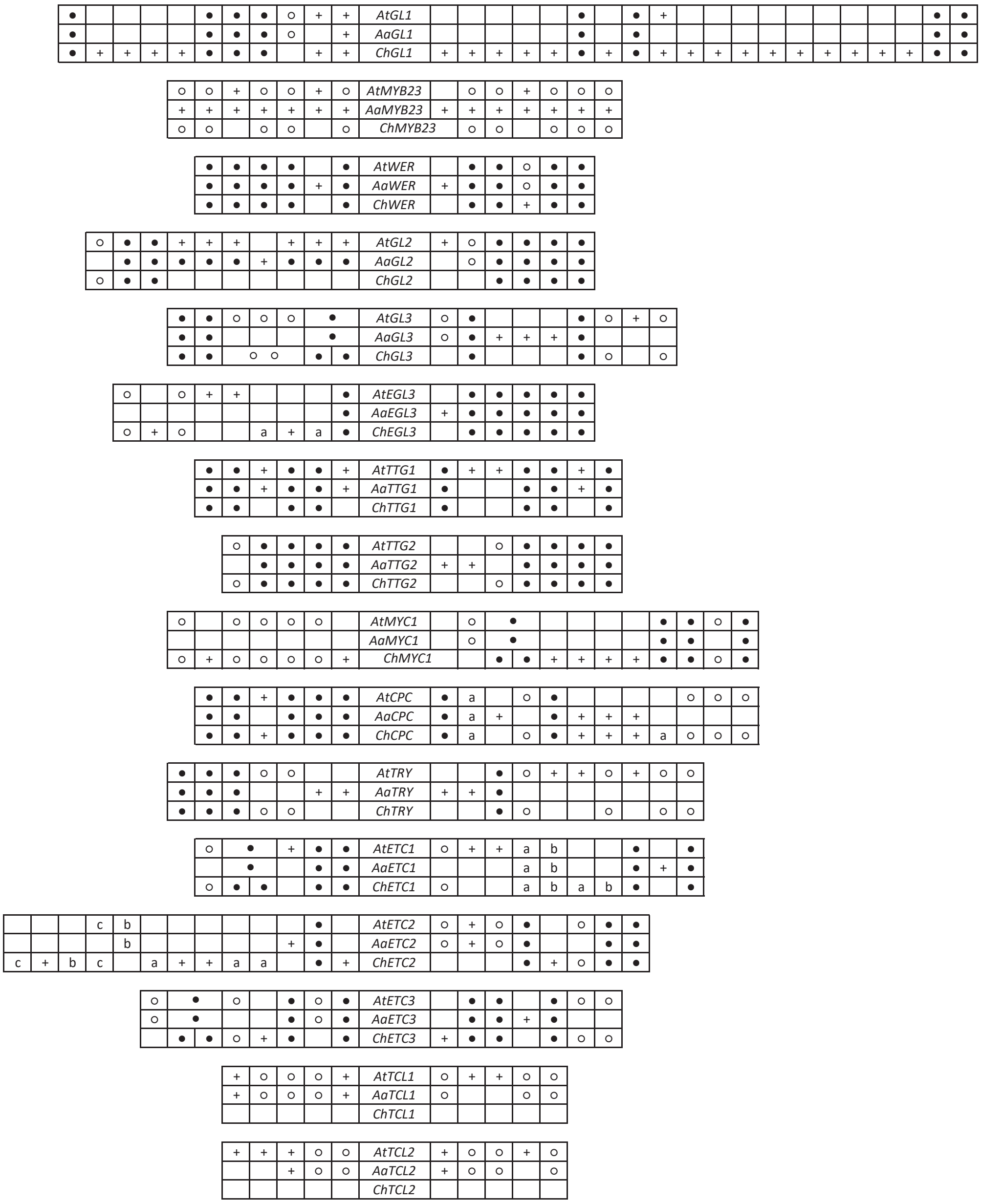

Figure S1. Synteny of all patterning genes in *A. thaliana*, *A. alpina*, and *C. hirsuta*. Because the three species are closely related, it is possible to identify ortholog genes based on the arrangement of neighboring genes. Each column represents one gene locus. Filled circles display gene orthologs in all three species, empty circles in two species. Pluses indicate additional genes and same letters indicate same genes. Merged cells mean that two or more loci correspond to one gene.

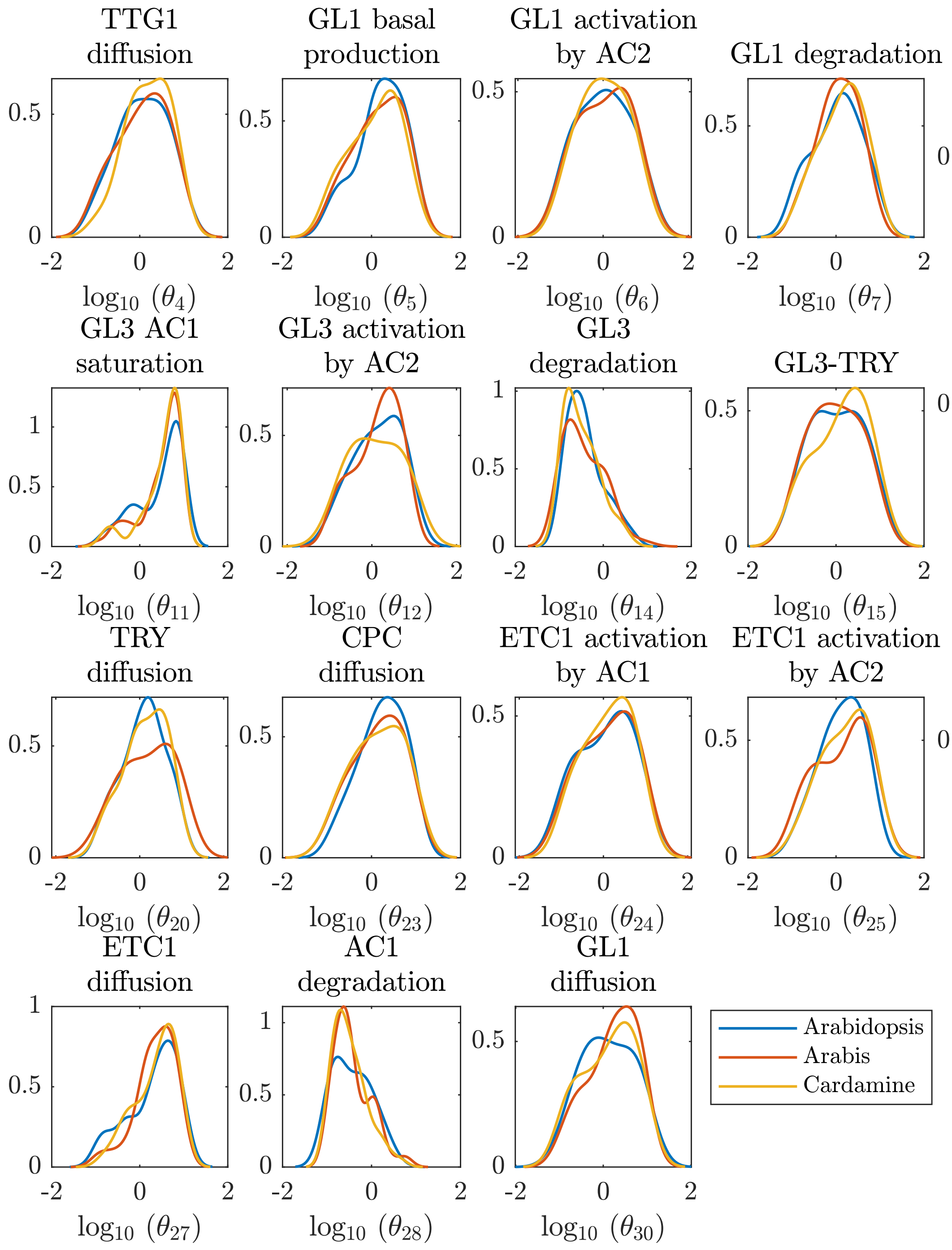

Figure S2. Parameter profile densities. Parameter distributions for which no significant difference between the species according to a Kolmogorov-Smirnov test, obtained from fitting the model output to the qPCR data.

2 SUPPLEMENTARY TABLES

Table S1. Summary of all Cq values genes Listed are all Cq values of the reference genes and investigated patterning genes in *A. thaliana*, *A. alpina*, and *C. hirsuta*

	Replicate	Reference genes		Genes of interest														
Arabidopsis thaliana		*18S rRNA*	*EF1 α*	*GL1*	*GL2*	*GL3*	*EGL3*	*TTG1*	*TTG2*	*MYC1*	*MYB23*	*CPC*	*TRY*	*ETC1*	*ETC2*	*ETC3*	*TCL1*	*TCL2*
	1.1	9.594	15.664	22.280	21.307	24.992	23.868	19.534	23.106	23.438	22.184	22.615	23.657	22.800	28.174	22.920	24.199	21.068
	1.2	9.285	15.435	22.379	21.351	25.124	23.845	19.321	23.400	23.369	22.452	22.795	24.051	22.762	31.964	22.855	23.949	21.099
	1.3	9.475	15.341	22.244	21.708	25.222	23.827	19.347	23.113	23.539	22.697	22.619	23.960	22.815	29.763	22.877	24.209	21.090
	2.1	9.127	15.122	22.642	21.451	25.139	23.945	19.562	23.271	23.720	22.851	22.804	24.056	23.122	27.391	23.424	24.645	21.617
	2.2	9.096	15.287	22.593	21.283	24.887	24.072	19.563	23.755	23.518	22.772	22.863	24.054	23.058	28.200	23.282	24.793	21.532
	2.3	8.959	15.455	22.844	21.405	25.172	24.208	19.372	23.177	23.455	22.836	23.097	24.247	23.041	27.134	23.342	24.786	21.553
	3.1	9.208	15.010	22.426	20.989	24.892	23.599	19.245	22.949	23.393	22.335	22.663	23.327	22.749	28.028	22.683	24.361	21.280
	3.2	9.240	15.162	22.025	21.348	24.991	23.604	18.865	23.027	23.026	22.120	22.694	23.443	22.546	28.655	22.563	24.305	21.264
	3.3	9.340	15.323	22.341	21.165	24.917	23.673	19.075	23.128	23.272	22.442	22.593	24.256	22.531	27.978	22.579	24.247	21.378
Arabis alpina		*RAN3*	*PP2A*	*GL1*	*GL2*	*GL3*	*EGL3*	*TTG1*	*TTG2*	*MYC1*	*MYB23*	*CPC*	*TRY*	*ETC1*	*ETC2*	*ETC3*	*TCL1*	*TCL2*
	1.1	15.410	19.341	19.681	20.210	19.933	21.844	18.264	20.260	19.160	19.374	19.809	17.913	21.410	23.799	18.967	23.821	23.112
	1.2	15.478	19.579	19.486	20.527	20.411	21.847	18.322	20.378	19.391	19.707	19.691	18.277	21.275	23.856	18.798	23.876	23.259
	1.3	15.242	19.311	19.666	20.851	20.236	22.448	18.327	20.394	19.513	19.507	19.860	18.241	21.582	24.178	19.428	24.058	23.229
	2.1	15.374	19.185	19.860	21.214	20.346	22.307	18.075	20.406	19.107	19.673	19.986	18.666	21.488	24.761	19.531	24.833	24.416
	2.2	15.160	19.229	19.833	21.144	20.431	22.323	17.702	20.458	19.198	19.935	19.840	18.835	21.555	24.883	19.340	24.902	24.476
	2.3	14.754	19.143	19.664	20.995	20.380	22.337	18.340	20.416	19.275	19.821	19.829	18.778	21.646	24.992	19.483	25.174	24.525
	3.1	15.488	18.936	19.378	20.190	20.222	22.270	18.131	19.923	18.969	19.190	19.770	18.185	21.083	23.109	19.221	23.391	22.890
	3.2	15.445	19.278	19.413	20.326	20.177	22.141	18.038	20.007	18.887	18.938	19.384	17.864	20.933	23.265	18.899	23.457	22.644
	3.3	15.239	19.245	19.358	20.618	19.909	22.230	17.834	19.952	18.993	19.185	19.402	17.943	20.920	23.136	18.664	23.472	22.785
Cardamine hirsuta		*GAPDH*	*TIP41*	*GL1*	*GL2*	*GL3*	*EGL3*	*TTG1*	*TTG2*	*MYC1*	*MYB23*	*CPC*	*TRY*	*ETC1*	*ETC2*	*ETC3*		
	1.1	14.861	18.789	23.876	20.523	20.364	19.996	19.894	21.357	21.400	20.999	23.560	21.231	19.895	22.623	21.247		
	1.2	14.890	18.862	23.666	20.573	20.490	20.452	19.649	21.519	21.500	21.239	23.254	21.322	19.872	22.765	21.345		
	1.3	14.618	19.034	23.656	20.507	20.618	14.858	19.622	21.439	21.627	21.302	23.423	21.376	20.042	20.768	21.368		
	2.1	15.370	19.127	24.866	21.542	21.775	21.408	19.966	22.569	22.039	23.152	24.382	22.388	21.925	23.113	23.130		
	2.2	15.426	19.147	24.665	21.369	21.906	21.481	19.911	22.695	21.949	23.315	24.245	22.358	21.982	23.124	22.919		
	2.3	15.467	19.119	24.693	21.413	21.921	21.426	20.015	22.559	22.074	23.295	24.223	22.339	22.063	23.125	22.917		
	3.1	15.991	18.917	24.955	21.040	22.914	21.846	19.878	23.147	22.461	22.828	24.537	22.618	22.549	22.722	22.746		
	3.2	15.796	18.947	24.850	21.328	22.920	22.003	19.943	23.094	22.571	22.809	24.446	22.555	22.653	22.722	22.723		
	3.3	15.992	19.082	24.892	20.935	22.925	21.994	19.922	23.219	22.547	23.037	24.245	22.584	22.563	22.797	22.661		

Table S2. Overview of parameters in the model given in equations (4) - (12) and their biological interpretation.

Parameter	Interpretation
θ_1	TTG1 basal production
θ_2	TTG1 degradation
θ_3	TTG1-GL3 binding
θ_4	TTG1 diffusion
θ_5	GL1 basal production
θ_6	GL1 activation by AC2
θ_7	GL1 degradation
θ_8	GL1-GL3 binding
θ_9	GL3 basal production
θ_{10}	GL3 activation by AC1
θ_{11}	Saturation of GL3 AC1 activation
θ_{12}	GL3 activation by AC2
θ_{13}	Saturation of GL3 AC2 activation
θ_{14}	GL3 degradation
θ_{15}	GL3-TRY binding
θ_{16}	GL3-CPC binding
θ_{17}	GL3-ETC binding
θ_{18}	TRY activation by AC1
θ_{19}	TRY degradation
θ_{20}	TRY diffusion
θ_{21}	CPC activation by AC2
θ_{22}	CPC degradation
θ_{23}	CPC diffusion
θ_{24}	ETC activation by AC1
θ_{25}	ETC activation by AC2
θ_{26}	ETC degradation
θ_{27}	ETC diffusion
θ_{28}	AC1 degradation
θ_{29}	AC2 degradation
θ_{30}	GL1 diffusion
θ_{31}	GL3 diffusion
